# Integrative Analysis Identified *IRF6* and *NDST1* as Potential Causal Genes for Ischemic Stroke

**DOI:** 10.3389/fneur.2019.00517

**Published:** 2019-05-15

**Authors:** Xing-Bo Mo, Shu-Feng Lei, Yong-Hong Zhang, Huan Zhang

**Affiliations:** ^1^Jiangsu Key Laboratory of Preventive and Translational Medicine for Geriatric Diseases, Soochow University, Suzhou, China; ^2^Center for Genetic Epidemiology and Genomics, School of Public Health, Soochow University, Suzhou, China; ^3^Department of Epidemiology, School of Public Health, Soochow University, Suzhou, China

**Keywords:** stroke, m^6^A, methylation, genome-wide association study, Mendelian randomization

## Abstract

**Objective:** To highlight potential functional variants and causal genes for ischemic stroke (IS) in genomic loci identified by genome-wide association studies (GWAS).

**Methods:** We examined the association between m^6^A-SNPs and IS in large scale GWAS. Furthermore, eQTL analysis was performed to evaluate the effect of m^6^A-SNPs on gene expression. The top associations between m^6^A-SNPs and gene expressions were validated in 40 individuals from the Chinese Han population. Besides, we applied differential expression analysis and Mendelian randomization (MR) analysis to detect potential causal genes for IS.

**Results:** We found 310 (7.39%) m^6^A-SNPs which were nominally associated with IS. The proportion of m^6^A-SNPs with *P* < 0.05 for IS was significantly higher than the non-m^6^A-SNPs (95%CI: [5.84%, 7.36%], *P* = 0.02). We found that the IS-associated m^6^A-SNP rs2013162 was associated with *IRF6* expression (*P* = 6.30 × 10^−23^), meanwhile *IRF6* was differentially expressed between IS cases and controls (*P* = 6.15 × 10^−3^) and showed a causal association with IS (*P* = 3.64 × 10^−4^). Similar results were found for m^6^A-SNP rs2273235 in the *NDST1* gene which was associated with cardioembolic stroke (*P* = 8.47 × 10^−3^). The associations of rs2013162 and rs2273235 with the expression of *IRF6* and *NDST1* were validated in blood cells (*P* = 0.0247 and 0.0007), respectively.

**Conclusions:** This study showed that m^6^A-SNPs may affect IS risk through altering gene expressions. The results suggested that m^6^A might play a role in IS etiology and gene expressions that affected by m^6^A may be causal factors for IS.

## Introduction

Ischemic stroke (IS) is the second leading cause of death worldwide (1). As a complex disease, genetic and epigenetic factors play important roles in IS etiology. Several genome-wide association studies (GWAS) have successfully identified many loci for IS and specific subtypes, including large artery stroke (LAS), cardioembolic stroke (CES), and small vessel stroke (SVS). A recent large-scale meta-analysis of GWAS in 521,612 individuals confirmed 32 IS-associated loci which offering mechanisms not previously implicated in stroke pathophysiology (2). Although previous GWAS have revolutionized the understanding of the genetic architecture of IS and provided a framework for prioritization of stroke risk variants and genes for further functional and experimental follow-up, identification of functional variants and causal genes in the GWAS loci is not finished but still a major challenge.

N^6^-methyladenosine (m^6^A) is a pervasive RNA modification that plays critical roles in mRNA stability, protein expression and several other cellular processes (3). Dysregulated m^6^A has been linked to cell fate during the endothelial-to-hematopoietic transition (4), cardiac homeostasis (5, 6) and brain diseases (7). Genetic variants, i.e., the m^6^A-associated SNPs (m^6^A-SNPs), can influence m^6^A by changing the RNA sequences of the target sites (8). If m^6^A modification was affected by this kind of variants, the biological process would likely be modified, leading to under-/overexpression of the protein (8).

Evaluation of the effect of genetic variants on m^6^A modification will increase our understanding of the pathogenic molecular mechanisms and uncover new causal variants. Until now, the relationship between m^6^A-SNPs and IS has not yet been clearly defined. Besides, determination of the association between m^6^A and IS in large sample at genome-wide scale in a large sample is hard to achieve nowadays. In this study we investigated the effect of the m^6^A-SNPs on IS and showed that by using the GWAS identified IS-associated m^6^A-SNPs as a bridge we can assess the relationship between m^6^A and IS indirectly. Meanwhile, we highlight some potential causal genetic variants and genes for IS.

## Materials and Methods

### Identification of m^6^A-SNPs for IS

In this study, we first investigated the effect of the m^6^A-SNPs on IS in the published summary data of a large scale GWAS (2). This GWAS comprised 521,612 individuals. Raw data used in the present analysis was the downloaded summary results from the initial GWAS, which included association *P* values of almost 8 million SNPs and indels for any IS (AIS) and common etiological subtypes of LAS, CES, and SVS. These datasets were available at the MEGASTROKE website (http://megastroke.org/).

To screen out the m^6^A-SNPs in these 8 million SNPs, we annotated them using a list of m^6^A-SNPs which were downloaded from the m6AVar database (http://m6avar.renlab.org/). The list contains 13,703 high, 54,222 medium and 284,089 low confidence level m^6^A-SNPs for human (8). After annotation of the SNPs in the GWAS summary dataset by the list of m^6^A-SNPs, we identified the m^6^A-SNPs which were associated with IS. Those m^6^A-SNPs with *P* < 0.05 were considered in the following analyses.

Among IS-associated SNPs, we determined if m^6^A-SNPs were overrepresented compared to what would be expected by chance. We randomly sampled 1,000 sets of non-m^6^A-SNPs (the same number of m^6^A-SNPs) from the GWAS datasets for IS as matched background, and then determined if the proportion of m^6^A-SNPs with *P* < 0.05 was significantly higher than the proportion of non-m^6^A-SNPs with *P* < 0.05 in the 1,000 sets for each trait. Besides, to assure that allele frequency differences between m^6^A and non-m^6^A-SNPs are not driving the conclusion that there is overrepresentation of m^6^A-SNPs in stroke cases, we selected from allele frequency bins of 1–5, 5–10, 11–20, 21–30, 31–40, and 41–50% for each set of non-m^6^A-SNPs to more precisely mirror the distribution of m^6^A-SNPs.

### eQTL Analysis

The m^6^A-SNPs may participate in gene expression regulation through exerting influence on RNA modification, thus they may be associated with gene expression level. We carried out the *cis*-acting eQTL analysis to obtain evidence on associations between the identified m^6^A-SNPs and gene expressions in HaploReg (https://pubs.broadinstitute.org/mammals/haploreg/haploreg.php). To validate the eQTLs we also tested genotypes and mRNA expression levels in peripheral blood mononuclear cells (PBMCs) of 40 unrelated Chinese Han individuals (age range from 27 to 67) using RT-PCR method to obtain additional evidence to support the top IS-associated m^6^A-SNPs. PBMCs were isolated from 15 ml peripheral blood by density gradient centrifugation using Lymphoprep (Sigma, life science, USA). Total RNA and DNA were extracted in the same lab according to the instructions recommended by the manufacturer. The study was approved by the ethical committee of Soochow University. The written informed consent was obtained from all of the participants.

### Differential Expression Analysis

We further tried to determine if the expression levels of genes which the IS-associated m^6^A-SNPs showed *cis*-eQTL effects on were associated with IS based on the expression profile data (GSE22255) available in the GEO database (http://www.ncbi.nlm.nih.gov/geo). GSE22255 contained data of gene expression levels in PBMCs from 20 IS cases to 20 controls (9). Differential expression was tested by comparing mean gene expression signals between cases and controls using *t*-test. The significance level of *P* = 0.05 was used for the differential expression analyses.

### Mendelian Randomization Analysis

We supposed that the IS-associated m^6^A-SNPs affect gene expression and consequently cause IS. To obtain additional evidence to support this idea, we conducted a summary data–based Mendelian randomization (SMR) analysis (10). SMR applies the principles of MR (11, 12) to jointly analyze eQTL and GWAS summary statistics in order to test for association between gene expression and a trait due to a shared variant at a locus. A HEIDI (heterogeneity in dependent instruments) test for heterogeneity in the resulting association statistics was performed. *P*_HEIDI_ > 0.05 means that there was no significant heterogeneity underlying the eQTL signals. The SMR software was downloaded from http://cnsgenomics.com/software/smr/. Genotype data of HapMap r23 CEU was used as a reference panel to calculate the linkage disequilibrium correlation for SMR analysis. The eQTL summary data from four studies were used in our SMR analysis. Westra et al. performed the largest eQTL meta-analysis so far in peripheral blood samples of 5,311 European healthy individuals (13). The genetic architecture of gene expression (GAGE) study detected eQTLs in peripheral blood in 2,765 European individuals (14). The cis-eQTL summary data from the GTEx whole blood (15) and brain (16) were used. Only SNPs within 1 Mb of the transcription start site are available for these two GTEx datasets.

## Results

### IS-associated m^6^A-SNPs

We found about 4,000 m^6^A-SNPs in each the GWAS datasets for AIS, LAS, CES, and SVS. Among these SNPs, 310 (7.39%), 305 (6.72%), 279 (6.18%), and 205 (6.26%) were nominally (*P* < 0.05) associated with AIS, LAS, CES, and SVS, respectively. The proportion of m^6^A-SNPs which have GWAS *P* < 0.05 for AIS was significantly higher than the non-m^6^A-SNPs (95%CI: [5.84%, 7.36%], *P* = 0.02). But it's not for LAS (95%CI: [5.55%, 6.94%], *P* = 0.09), CES (95%CI: [5.45%, 6.82%], *P* = 0.45), or SVS (95%CI: [5.10%, 6.84%], *P* = 0.26), which were analyzed in smaller sample than AIS. In the original METASTROKE GWAS, SNPs with minor allele frequencies (MAF) ≥ 0.01 were examined. Therefore, the MAF of SNPs considered in our study was ≥0.01. In fact, the MAF of about 90% of the 321 IS-associated SNPs were >0.05. The distributions of MAF were not different between m^6^A-SNPs and non-m^6^A-SNPs with *P* < 0.05 (goodness-of-fit test *P* = 0.1551). While selected from allele frequency bins of 1–5% (number of SNPs sampled *n* = 583), 6–10% (*n* = 663), 11–20% (*n* = 1,064), 21–30% (*n* = 720), 31–40% (*n* = 628), and 41–50% (*n* = 539) for each of the 1,000 sets of non-m^6^A-SNPs to mirror the distribution of m^6^A-SNPs for AIS, we also found that the proportion of m^6^A-SNPs with *P* < 0.05 was significantly higher than the non-m^6^A-SNPs (95%CI: [5.89%, 7.29%], *P* = 0.014).

**Figure 1:**
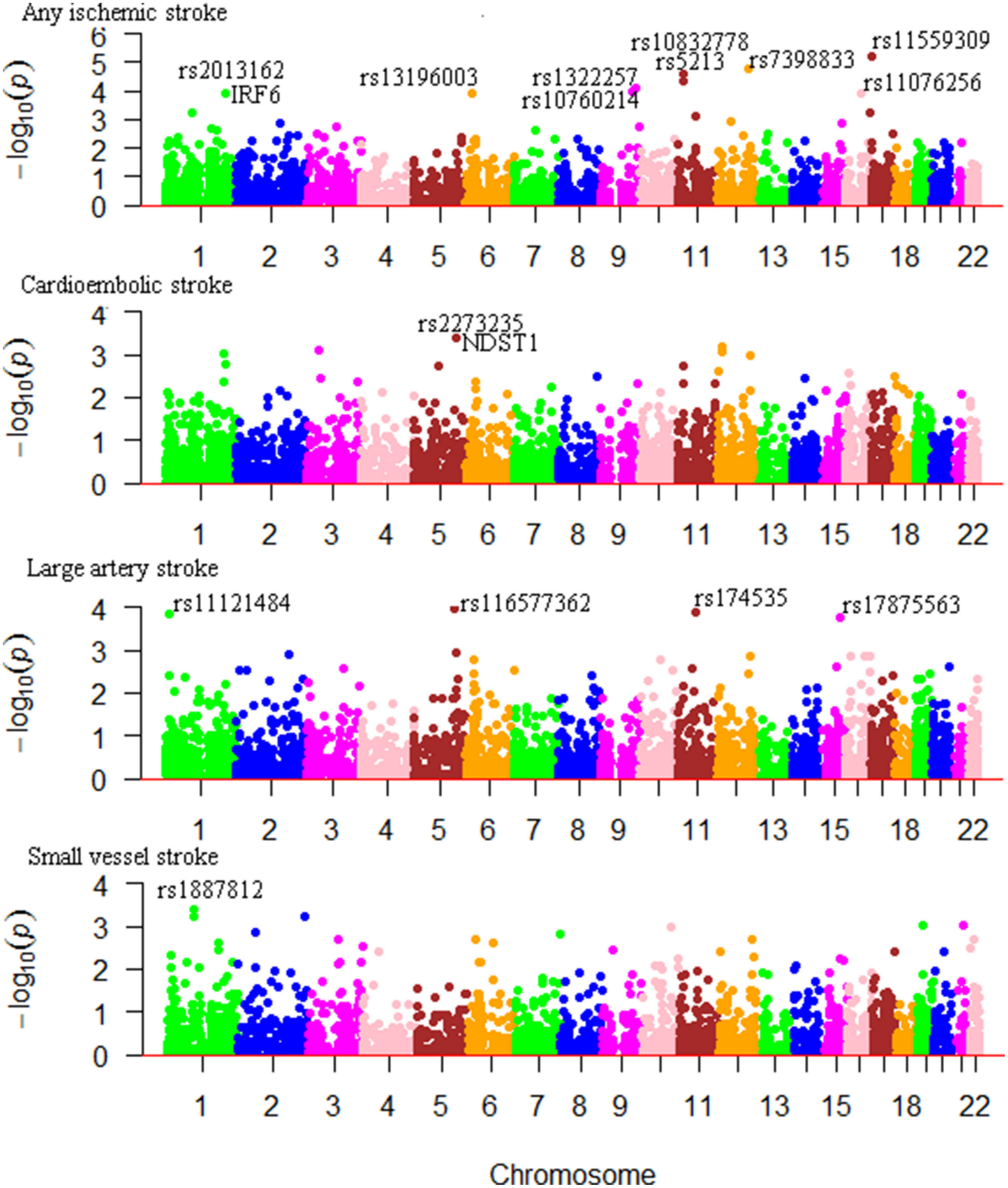
Genome-wide results for the association between m^6^A-SNPs and IS. The Manhattan plots show –log_10_*P* values for the m^6^A-SNPs associated with IS and subtypes. The data was from the IS GWAS published by the MEGASTROKE consortium at 2018 (2).

We considered Bonferroni-correction for the results (*P* < 0.05/4000 for roughly 4000 SNPs). The association between m^6^A-SNPs rs11559309 (*PRPF8, P* = 6.18 × 10^−6^) and AIS reached the significance level of 1.25 × 10^−5^, followed by rs7398833 (*P* = 1.78 × 10^−5^) in *CUX2* gene, rs5213 (*P* = 2.46 × 10^−5^) in *KCNJ11* gene and rs10832778 (*P* = 4.25 × 10^−5^) in *NCR3LG1* gene. The most significant m^6^A-SNPs for LAS, CES, and SVS were rs174535 (*P* = 6.05 × 10^−5^) in *MYRF* gene, rs2273235 (*P* = 3.99 × 10^−4^) in *NDST1* gene and rs1887812 (*P* = 4.05 × 10^−4^) in *BRDT* gene (Table 1; Figure 1), respectively.

**Table 1:** The identified m^6^A-SNPs for ischemic stroke.

**SNP**	**CHR**	**SNP position******	**MAF**	**Gene**	**Mutation Type**	***P*****-value≠**					**m^**6**^A_ID**	**m^**6**^A position******	**m^**6**^A function**
						**TRAN**	**EURO**	**eQTL**	**DEG**	**SMR**			
**AIS**													
rs2013162	1	209968684	0.3736	*IRF6*	Syn	1.27E-04	6.73E-04	6.30E-23	6.15E-03	3.64E-04	m6A_ID_132331	209968661	Loss
rs13196003	6	24535999	0.0725	*ALDH5A1*	UTR3	1.29E-04	3.36E-04				m6A_ID_189376	24535978	Loss
rs1322257	9	114480562	0.0112	*C9orf84*	Nonsyn	1.02E-04	8.28E-03				m6A_ID_226979	114480545	Gain
rs10760214	9	125002246	0.4763	*RBM18*	UTR3	7.54E-05	6.38E-04	1.29E-35		2.44E-03	m6A_ID_227927	125002247	Loss
rs10832778	11	17394073	0.3885	*NCR3LG1*	UTR3	4.25E-05	3.97E-04	1.18E-17			m6A_ID_244519	17394071	Gain
rs5213	11	17408404	0.3615	*KCNJ11*	UTR3	2.46E-05	4.49E-04	7.57E-13		7.21E-04	m6A_ID_27449	17408423	Loss
rs7398833	12	111786892	0.2509	*CUX2*	UTR3	1.78E-05	2.46E-05		1.51E-02		m6A_ID_265695	111786917	Gain
rs11076256	16	58752466	0.0818	*GOT2*	Nonsyn	1.29E-04	3.46E-03				m6A_ID_43360	58752472	Loss
rs11559309	17	1556911	0.0374	*PRPF8*	Syn	6.18E-06	7.83E-06				m6A_ID_301320	1556907	Gain
**LAS**													
rs11121484	1	9784423	0.0769	*PIK3CD*	Syn	1.40E-04	1.18E-02		4.20E-02		m6A_ID_112617	9784425	Gain
rs116577362	5	140242897	0.0215	*PCDHA*	Nonsyn	1.04E-04	1.04E-04				m6A_ID_184329	140242899	Loss
rs174535	11	61551356	0.3392	*MYRF*	Nonsyn	1.31E-04	6.05E-05				m6A_ID_247771	61551331	Gain
rs17875563	15	81604293	0.2405	*IL16*	UTR3	1.67E-04	1.83E-03	9.52E-09		3.97E-02	m6A_ID_287576	81604291	Gain
**CES**													
rs2273235	5	149907533	0.4821	*NDST1*	Syn	3.99E-04	1.04E-03	3.23E-07	2.65E-02	8.47E-03	m6A_ID_185695	149907528	Loss
**SVS**													
rs1887812	1	92414993	0.2074	*BRDT*	UTR5	4.05E-04	8.51E-04				m6A_ID_121270	92414991	Loss

*AIS, Any ischemic stroke; CES, Cardioembolic stroke; CHR, Chromosome; DEG, Differential expression gene; eQTL, Expression quantitative trait; LAS, Large artery stroke; MAF, Minor allele frequency (in Europeans); Nonsyn, Nonsynonymous; SMR, Summary data based Mendelian randomization; SNP, Single nucleotide polymorphism; SVS, Small vessel stroke; Syn, Synonymous; UTR, Untranslated region. †Assembly: GRCh37.p13. ≠TRAN: stroke association P values based-on trans ethnic data; EURO, Stroke association P-values based-on European-only data*.

### eQTL Analysis

To further clarify the possible functional mechanisms underlying the identified m^6^A-SNPs in association with IS, we investigated whether they were associated with gene expression levels. In total, 6 of the 15 IS-associated m^6^A-SNPs showed *cis*-eQTL signals with the 6 local genes in different cells or tissues (Table 1; Table S1). The 5 IS-associated m^6^A-SNPs (except rs10832778) might alter regulatory motifs (17) (Table S1). Besides, rs7398833 (*CUX2*) was associated with expression of *ALDH2* (*P* = 1.47 × 10^−5^), *ATXN2* (*P* = 2.74 × 10^−6^), *FAM109A* (*P* = 3.41 × 10^−3^) and *SH2B3* (*P* = 1.27 × 10^−10^), and rs174535 (*MYRF*) was associated with expression of *FADS1* (*P* = 2.37 × 10^−31^), *FADS2* (*P* = 6.68 × 10^−28^), and *TMEM258* (*P* = 1.00 × 10^−56^).

### Differential Expression Analysis

For the genes which were presented in Table 1, we compared mRNA expression signals in an expression study. In this expression dataset, we found that *IRF6, CUX2, PIK3CD* and *NDST1* were differentially expressed in PBMCs (*P* < 0.05) (Table 1). Among them, the expression levels of *IRF6* and *NDST1* were affected by rs2013162 (*P* = 6.30 × 10^−23^) and rs2273235 (*P* = 3.23 × 10^−7^) according to eQTL data from HaploReg, respectively. It means that these 2 m^6^A-SNPs may affect IS through altering the local gene.

### Mendelian Randomization Analysis

The SMR analysis identified several genes as potential causal genes underlying IS GWAS association (*P*_SMR_ < 5 × 10^−6^), and there was no significant heterogeneity underlying the eQTL signals (*P*_HEIDI_ > 0.05) (Table S2). For the 15 genes listed in Table 1, SMR tests (*P* < 3.33 × 10^−3^ were considered significant) identified that three genes (*IRF6, RBM18* and KCNJ11) were significantly associated with AIS, *IL16* was associated with LAS and *NDST1* was associated with CES (Table 1). All of these five genes passed the HEIDI tests (*P*_HEIDI_ > 0.05).

### The SNP-Expression-IS Trios

**Figure 2:**
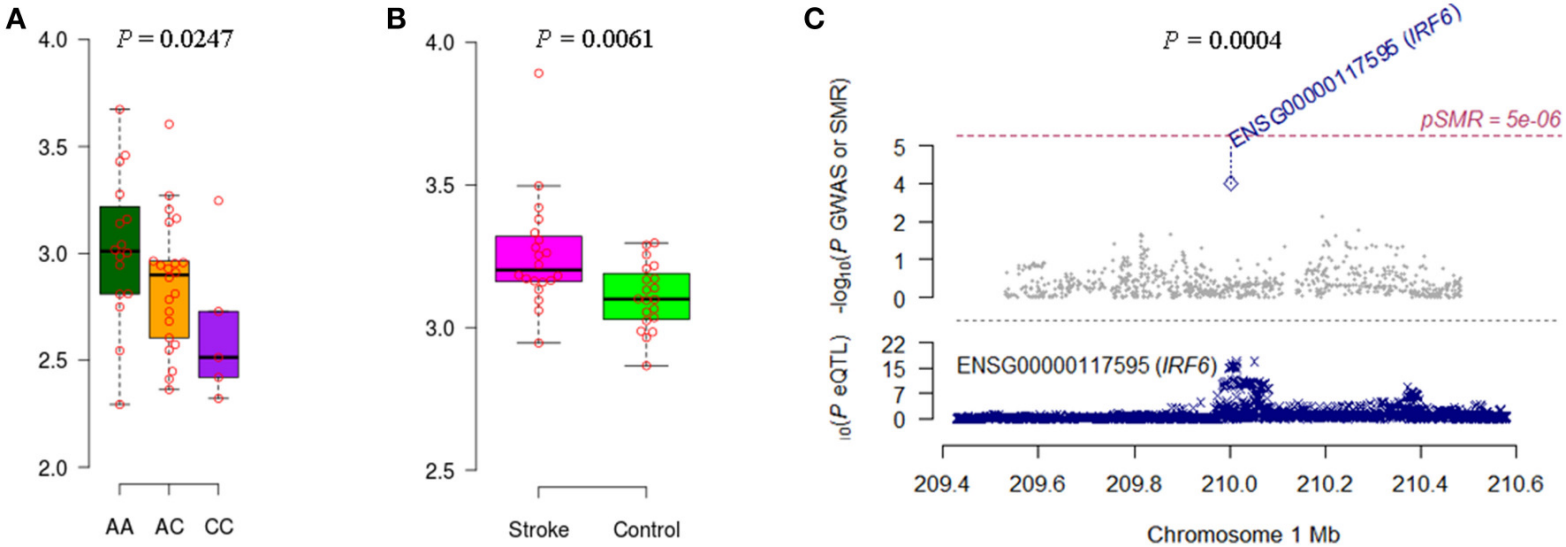
Association between rs2013162 and *IRF6* expression and AIS. **(A)** The minor allele C carriers of rs2013162 tend to have lower *IRF6* gene expression levels in PBMCs of Chinese individuals. **(B)** *IRF6* was differentially expressed between IS cases and controls according to the data of GSE22255. **(C)** *IRF6* expression seems to be causally associated with AIS (*P*_SMR_ = 3.64 × 10^−4^, *P*_HEIDI_ = 0.11).

**Figure 3:**
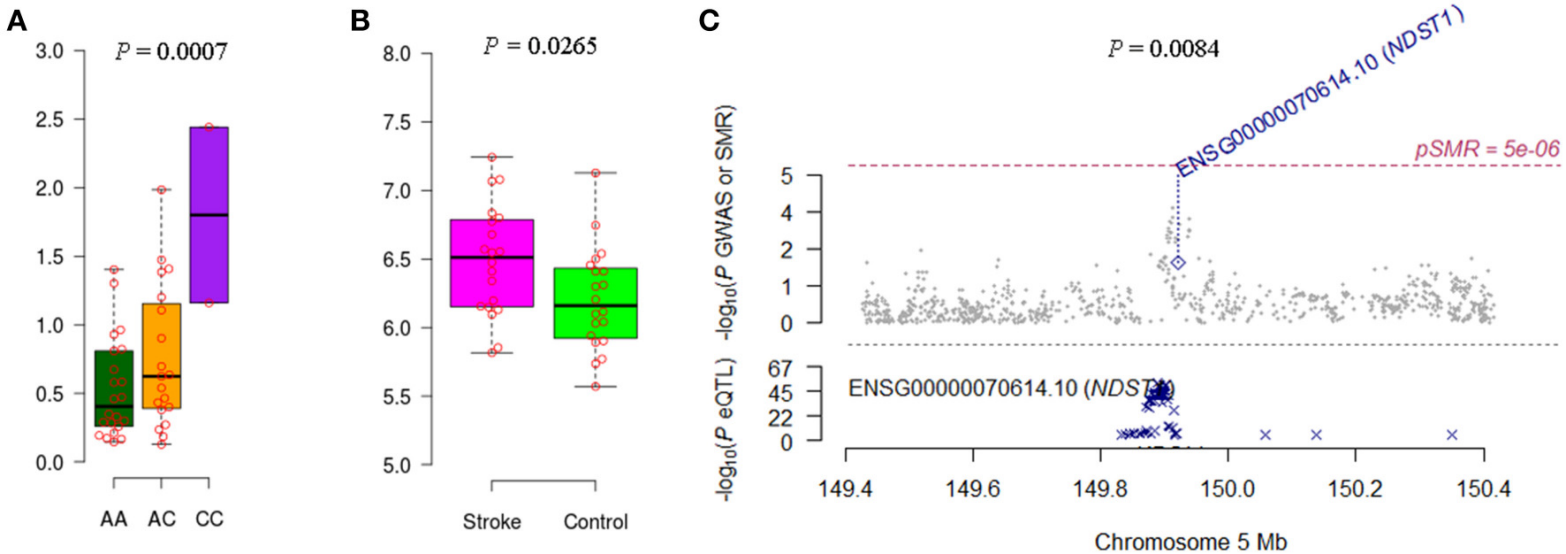
Association between rs2273235 and *NDST1* expression and CES. **(A)** The minor allele C carriers of rs2273235 tend to have higher *NDST1* gene expression levels in PBMCs of Chinese individuals. **(B)** *NDST1* was differentially expressed between IS cases and controls according to the data of GSE22255. **(C)** *NDST1* expression seems to be causally associated with CES (*P*_SMR_ = 8.47 × 10^−3^, *P*_HEIDI_ = 0.65).

We noticed that *IRF6* and *NDST1* showed more convincing evidence. For example, the *IFR6* SNPs achieved suggestive evidence of association with AIS (*P* < 5 × 10^−5^) (Figure S1). The AIS-associated (*P* = 1.27 × 10^−4^) m^6^A-SNP rs2013162 may affect *IRF6* expression (*P* = 6.30 × 10^−23^), *IRF6* was differentially expressed between IS cases and controls (*P* = 6.15 × 10^−3^) and showed a causal association with AIS (*P*_SMR_ = 3.64 × 10^−4^, *P*_HEIDI_ = 0.11). The association between *NDST1* SNPs and CES did not reach the genome-wide significance level but many SNPs that were in linkage disequilibrium showed suggestive evidence (Figure S2). The CES-associated (*P* = 3.99 × 10^−4^) m^6^A-SNP rs2273235 may affect *NDST1* expression (*P* = 3.23 × 10^−7^), *NDST1* was differentially expressed between IS cases and controls (*P* = 2.65 × 10^−2^) and showed a causal association with CES (*P*_SMR_ = 8.47 × 10^−3^, *P*_HEIDI_ = 0.65). So subsequently, we tested the association between rs2013162 and rs2273235 and mRNA expression levels of *IRF6* and *NDST1*, respectively. The MAF for rs2013162 and rs2273235 were 0.3736 and 0.4821 in Europeans (Table 1), and 0.3721 and 0.2674 in the Han Chinese population according to our 40 sample, respectively. These two SNPs were high-frequency variants in both of the European and East Asian populations. We validated that rs2013162 and rs2273235 were significantly associated with *IRF6* and *NDST1* expression levels in PBMCs from the Chinese Han individuals (linear regression *P* = 0.0247 and 0.0007) (Figures 2, 3), respectively.

## Discussion

This study represents the first effort to identify putative functional variants and causal genes for IS by integrating m^6^A-SNPs data, gene expression data and genetic association data from large scale GWAS. We found out several m^6^A-SNPs (e.g., rs2013162 and rs2273235) which may be putative functional variants for IS. Moreover, we also showed that these SNPs have potential to affect transcription regulation and were associated with expressions of the local genes (e.g., *IRF6* and *NDST1*). These genes may be potential causal genes for IS.

It seems that m^6^A-SNPs may be causally associated with IS by influencing gene expression. Studies have shown that m^6^A modification plays a pivotal role in the regulation of downstream molecular events such as nuclear export, stability, translatability, splicing, and miRNA processing (18). So one of the functional interpretations for the effect of m^6^A-SNPs on IS could be their influence on gene expression levels. Then if the m^6^A-SNP is causal SNP, the affected gene should be causal gene. In this study we detected several potential causal genes for IS based on large data of eQTL and GWAS. For these genes, we validated the association between m^6^A-SNPs and gene expression (e.g., *IRF6* and *NDST1*) in public and in-house data. This finding supported our hypothesis that m^6^A-SNPs may be causally associated with IS by influencing gene expression.

The *IRF6* gene encodes a member of the IRF family of transcription factors which play roles in cardiovascular diseases (19, 20). *IRF6* is likely to promote inflammation to *Porphyromonas gingivalis* through its regulation of IL-36γ (21), and exhibits tumor suppressor activity in squamous cell carcinomas (22). The RIPK4-IRF6 signaling axis plays a multifaceted role in barrier epithelial homeostasis through its regulation of both keratinocyte inflammation and differentiation (23). *IRF6* also contributes to host defense by providing specificity to the regulation of inflammatory chemokine expression by TLR2 in epithelial cells (24). The synonymous m^6^A-SNP rs2013162 in *IRF6* (1q32.2) has been well studied on disease. This SNP was not reported in the original METASTROKE GWAS (2). But by looking up the published GWAS data, we found that rs2013162 was also associated with intracerebral hemorrhage (*P* = 3.26 × 10^−4^) (25) and large artery atherosclerosis-related stroke (*P* = 9.34 × 10^−3^) (26).

*NDST1* encodes a bifunctional GlcNAc N-deacetylase/N-sulfotransferase with important functions in biosynthesis of heparan sulfate, which play roles in triglyceride-rich lipoprotein clearance (27), stroke (28, 29), and allergic airway inflammation through the regulation of recruitment of inflammatory cells to the airways by mediating interaction of leukocytes with the vascular endothelium (30). Suppression of *NDST1* in endothelial cells results in reduced responsiveness to *VEGFA* (31), which plays important role in cardiovascular diseases. The synonymous m^6^A-SNP rs2273235 in *NDST1* (5q33.1) was associated with CES in two GWAS (2, 32). This SNP has the potential to alter regulatory motifs Evi-1, NF-AT1 and PTF1-beta, and locates in a CpG island (CpG: 19) and near DNase I hypersensitive sites (Figure S3). All in all, the identified m^6^A-SNPs and genes can be suggested as important candidates for further genetic association and functional studies.

This study has several limitations. First, no genome-wide significant associations between m^6^A-SNPs and IS was found. The significant associations proposed in this paper were at the borderline level of statistical significance after multiple testing adjustments. Second, the sample size of the differential gene expression study was small, so very few associations between gene expression levels and IS have been identified. Third, only a very small proportion of m^6^A-SNPs were examined in this study. To fully recognize the impact of m^6^A-SNPs on IS, we still need to identify more m^6^A-SNPs (especially the rare variants) and the effects of the large amounts of m^6^A-SNPs on IS should be evaluated in larger GWAS datasets. Finally, the functionalities of the detected SNPs, especially effects on m^6^A modification, have not been experimentally validated. Further experiments are needed to determine their functions.

## Conclusions

In summary, the present study found out several IS-associated m^6^A-SNPs, and showed that these SNPs may affect the expressions of the local genes which might be potential causal genes for IS. This study increases our understanding on the regulation patterns of SNP. Although we found supplementary functional information to support the significant findings, further functional studies were needed to elucidate the mechanisms.

## Data Availability

All datasets generated for this study are included in the manuscript and/or the Supplementary Files.

## Ethics Statement

This study was carried out in accordance with the recommendations of Soochow University with written informed consent from all subjects. All subjects gave written informed consent in accordance with the Declaration of Helsinki. The protocol was approved by the Soochow University.

## Author Contributions

X-BM, S-FL, Y-HZ, and HZ contributed at all stages of manuscript preparation. HZ critically appraised and approved the final manuscript. All authors were involved in data recording and discussed the results.

### Conflict of Interest Statement

The authors declare that the research was conducted in the absence of any commercial or financial relationships that could be construed as a potential conflict of interest.

## References

[1] GBD 2016 Causes of Death Collaborators Global, regional, and national age-sex specific mortality for 264 causes of death, 1980–2016: a systematic analysis for the Global Burden of Disease Study 2016. Lancet. (2017) 390:1151–210. 10.1016/S0140-6736(17)32152-9 28919116 PMC5605883

[2] Malik R Chauhan G Traylor M Sargurupremraj M Okada Y Mishra A. Multiancestry genome-wide association study of 520,000 subjects identifies 32 loci associated with stroke and stroke subtypes. Nat Genet. (2018) 50:524–37. 10.1038/s41588-018-0058-3 29531354 PMC5968830

[3] Fu Y Dominissini D Rechavi G He C Gene expression regulation mediated through reversible m(6)A RNA methylation. Nat Rev Genet. (2014) 15:293–306. 10.1038/nrg3724 24662220

[4] Zhang C Chen Y Sun B Wang L Yang Y Ma D m(6)A modulates haematopoietic stem and progenitor cell specification. Nature. (2017) 549:273–6. 10.1038/nature23883 28869969

[5] Mathiyalagan P Adamiak M Mayourian J Sassi Y Liang Y Agarwal N FTO-dependent N(6)-methyladenosine regulates cardiac function during remodeling and repair. Circulation. (2019) 139:518–32. 10.1161/CIRCULATIONAHA.118.033794 29997116 PMC6400591

[6] Dorn LE Lasman L Chen J Xu X Hund TJ Medvedovic M The N(6)-methyladenosine mRNA methylase METTL3 controls cardiac homeostasis and hypertrophy. Circulation. (2019) 139:533–45. 10.1161/CIRCULATIONAHA.118.036146 30586742 PMC6340720

[7] Hess ME Hess S Meyer KD Verhagen LA Koch L Bronneke HS. The fat mass and obesity associated gene (Fto) regulates activity of the dopaminergic midbrain circuitry. Nat Neurosci. (2013) 16:1042–8. 10.1038/nn.3449 23817550

[8] Zheng Y Nie P Peng D He Z Liu M Xie Y. m6AVar: a database of functional variants involved in m6A modification. Nucleic Acids Res. (2018) 46:D139–D45. 10.1093/nar/gkx895 29036329 PMC5753261

[9] Krug T Gabriel JP Taipa R Fonseca BV Domingues-Montanari S Fernandez-Cadenas I. TTC7B emerges as a novel risk factor for ischemic stroke through the convergence of several genome-wide approaches. J Cereb Blood Flow Metab. (2012) 32:1061–72. 10.1038/jcbfm.2012.24 22453632 PMC3367223

[10] Zhu Z Zhang F Hu H Bakshi A Robinson MR Powell JE. Integration of summary data from GWAS and eQTL studies predicts complex trait gene targets. Nat Genet. (2016) 48:481–7. 10.1038/ng.3538 27019110

[11] Smith GD Ebrahim S. 'Mendelian randomization': can genetic epidemiology contribute to understanding environmental determinants of disease? Int J Epidemiol. (2003) 32:1–22. 10.1093/ije/dyg070 12689998

[12] Davey Smith G Hemani G Mendelian randomization: genetic anchors for causal inference in epidemiological studies. Hum Mol Genet. (2014) 23:R89–98. 10.1093/hmg/ddu328 25064373 PMC4170722

[13] Westra HJ Peters MJ Esko T Yaghootkar H Schurmann C Kettunen J. Systematic identification of trans eQTLs as putative drivers of known disease associations. Nat Genet. (2013) 45:1238–43. 10.1038/ng.2756 24013639 PMC3991562

[14] Lloyd-Jones LR Holloway A McRae A Yang J Small K Zhao J The genetic architecture of gene expression in peripheral blood. Am J Hum Genet. (2017) 100:228–37. 10.1016/j.ajhg.2016.12.008 28065468 PMC5294670

[15] Battle A Brown CD Engelhardt BE Montgomery SB. Genetic effects on gene expression across human tissues. Nature. (2017) 550:204–13. 10.1038/nature24277 29022597 PMC5776756

[16] Qi T Wu Y Zeng J Zhang F Xue A Jiang L. Identifying gene targets for brain-related traits using transcriptomic and methylomic data from blood. Nat Commun. (2018) 9:2282. 10.1038/s41467-018-04558-1 29891976 PMC5995828

[17] Kheradpour P Kellis M. Systematic discovery and characterization of regulatory motifs in ENCODE TF binding experiments. Nucleic Acids Res. (2014) 42:2976–87. 10.1093/nar/gkt1249 24335146 PMC3950668

[18] Visvanathan A Somasundaram K mRNA traffic control reviewed: N6-Methyladenosine (m(6) A) takes the driver's seat. Bioessays. (2018) 40:1700093 10.1002/bies.201700093 29205437

[19] Guo S Li ZZ Jiang DS Lu YY Liu Y Gao L. IRF4 is a novel mediator for neuronal survival in ischaemic stroke. Cell Death Differ. (2014) 21:888–903. 10.1038/cdd.2014.9 24510125 PMC4013523

[20] Szelag M Piaszyk-Borychowska A Plens-Galaska M Wesoly J Bluyssen HA. Targeted inhibition of STATs and IRFs as a potential treatment strategy in cardiovascular disease. Oncotarget. (2016) 7:48788–812. 10.18632/oncotarget.9195 27166190 PMC5217051

[21] Huynh J Scholz GM Aw J Kwa MQ Achuthan A Hamilton JA. IRF6 regulates the expression of IL-36gamma by human oral epithelial cells in response to *Porphyromonas gingivalis*. J Immunol. (2016) 196:2230–8. 10.4049/jimmunol.1501263 26819203

[22] Botti E Spallone G Moretti F Marinari B Pinetti V Galanti S. Developmental factor IRF6 exhibits tumor suppressor activity in squamous cell carcinomas. Proc Natl Acad Sci USA. (2011) 108:13710–5. 10.1073/pnas.1110931108 21807998 PMC3158164

[23] Kwa MQ Scholz GM Reynolds EC. RIPK4 activates an IRF6-mediated proinflammatory cytokine response in keratinocytes. Cytokine. (2016) 83:19–26. 10.1016/j.cyto.2016.03.005 27014863

[24] Kwa MQ Nguyen T Huynh J Ramnath D De Nardo D Lam PY. Interferon regulatory factor 6 differentially regulates Toll-like receptor 2-dependent chemokine gene expression in epithelial cells. J Biol Chem. (2014) 289:19758–68. 10.1074/jbc.M114.584540 24872416 PMC4094085

[25] Woo D Falcone GJ Devan WJ Brown WM Biffi A Howard TD. Meta-analysis of genome-wide association studies identifies 1q22 as a susceptibility locus for intracerebral hemorrhage. Am J Hum Genet. (2014) 94:511–21. 10.1016/j.ajhg.2014.02.012 24656865 PMC3980413

[26] International Stroke Genetics Consortium (ISGC) NSGNS. Loci associated with ischaemic stroke and its subtypes (SiGN): a genome-wide association study. Lancet Neurol. (2016) 15:174–84. 10.1016/S1474-4422(15)00338-5 26708676 PMC4912948

[27] Stanford KI Wang L Castagnola J Song D Bishop JR Brown JR. Heparan sulfate 2-O-sulfotransferase is required for triglyceride-rich lipoprotein clearance. J Biol Chem. (2010) 285:286–94. 10.1074/jbc.M109.063701 19889634 PMC2804175

[28] Khelif Y Toutain J Quittet MS Chantepie S Laffray X Valable S. A heparan sulfate-based matrix therapy reduces brain damage and enhances functional recovery following stroke. Theranostics. (2018) 8:5814–27. 10.7150/thno.28252 30613264 PMC6299437

[29] Hill JJ Jin K Mao XO Xie L Greenberg DA. Intracerebral chondroitinase ABC and heparan sulfate proteoglycan glypican improve outcome from chronic stroke in rats. Proc Natl Acad Sci USA. (2012) 109:9155–60. 10.1073/pnas.1205697109 22615373 PMC3384160

[30] Zuberi RI Ge XN Jiang S Bahaie NS Kang BN Hosseinkhani RM. Deficiency of endothelial heparan sulfates attenuates allergic airway inflammation. J Immunol. (2009) 183:3971–9. 10.4049/jimmunol.0901604 19710461 PMC2872128

[31] Kasza Z Fredlund Fuchs P Tamm C Eriksson AS O'Callaghan P Heindryckx F. MicroRNA-24 suppression of N-deacetylase/N-sulfotransferase-1 (NDST1) reduces endothelial cell responsiveness to vascular endothelial growth factor A (VEGFA). J Biol Chem. (2013) 288:25956–63. 10.1074/jbc.M113.484360 23884416 PMC3764800

[32] Malik R Traylor M Pulit SL Bevan S Hopewell JC Holliday EG. Low-frequency and common genetic variation in ischemic stroke: the METASTROKE collaboration. Neurology. (2016) 86:1217–26. 10.1212/WNL.0000000000002528 26935894 PMC4818561

