# Supplementary material for: Integrative Analysis Identified IRF6 and NDST1 as Potential Causal Genes for Ischemic Stroke
Source: Front Neurol. 2019 May 15;10:517. doi: 10.3389/fneur.2019.00517 (PMC6529957; doi:10.3389/fneur.2019.00517)
Supplement: Supplementary file 1 [file Presentation_1.pdf]

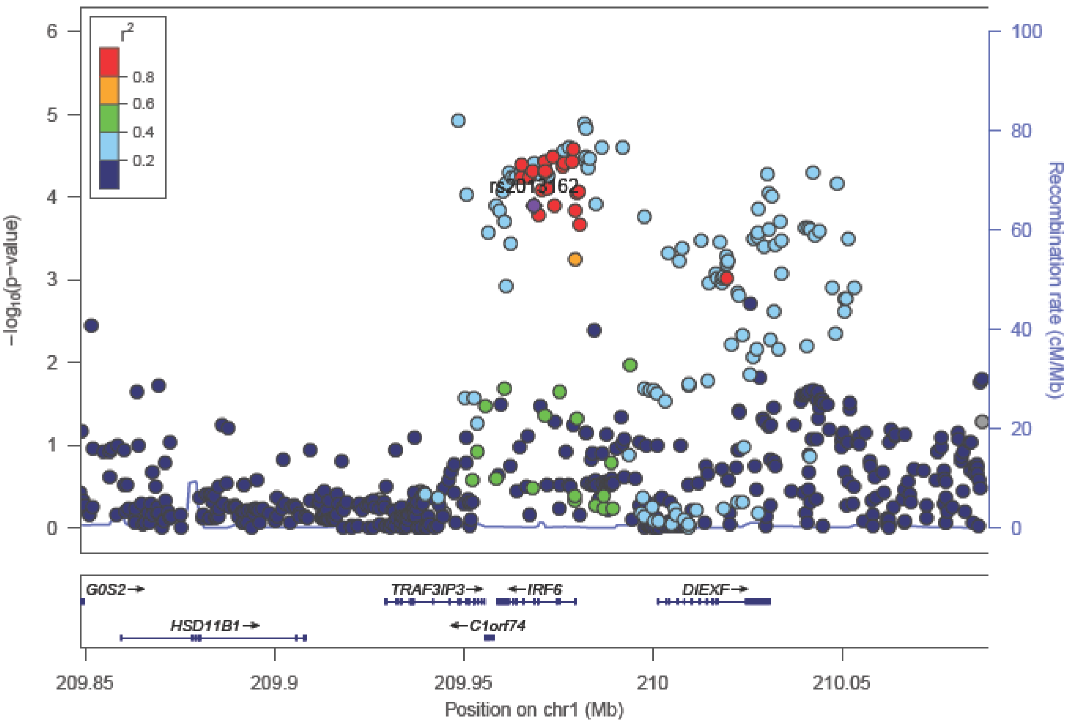

Figure S1 Regional plot for the association between *IFR6* SNPs and AIS. The *IFR6* SNPs achieved suggestive evidence of association with AIS ($P < 5 \times 10^{-5}$). This region (1q32.2) was not a GWAS reported AIS locus.

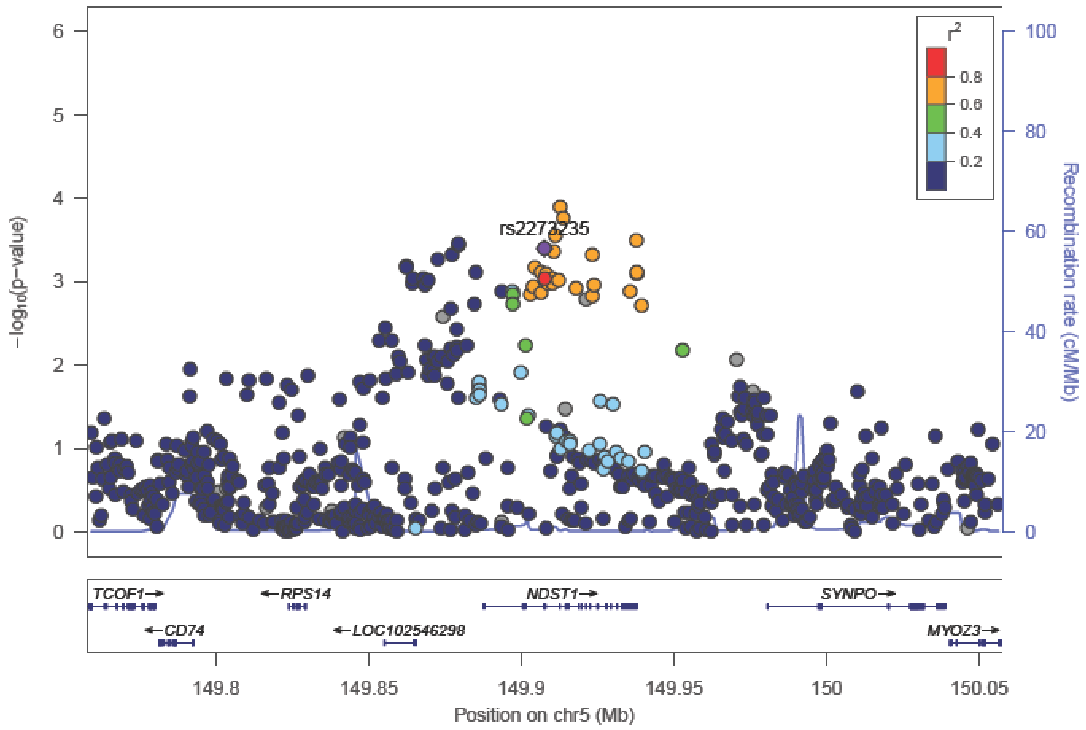

Figure S2 Regional plot for the association between *NDST1* SNPs and CES. This association did not reach the genome-wide significance level of 5×10^{-8} but many SNPs in linkage disequilibrium showed suggestive evidence. This region (5q33.1) was not a GWAS reported CES locus.

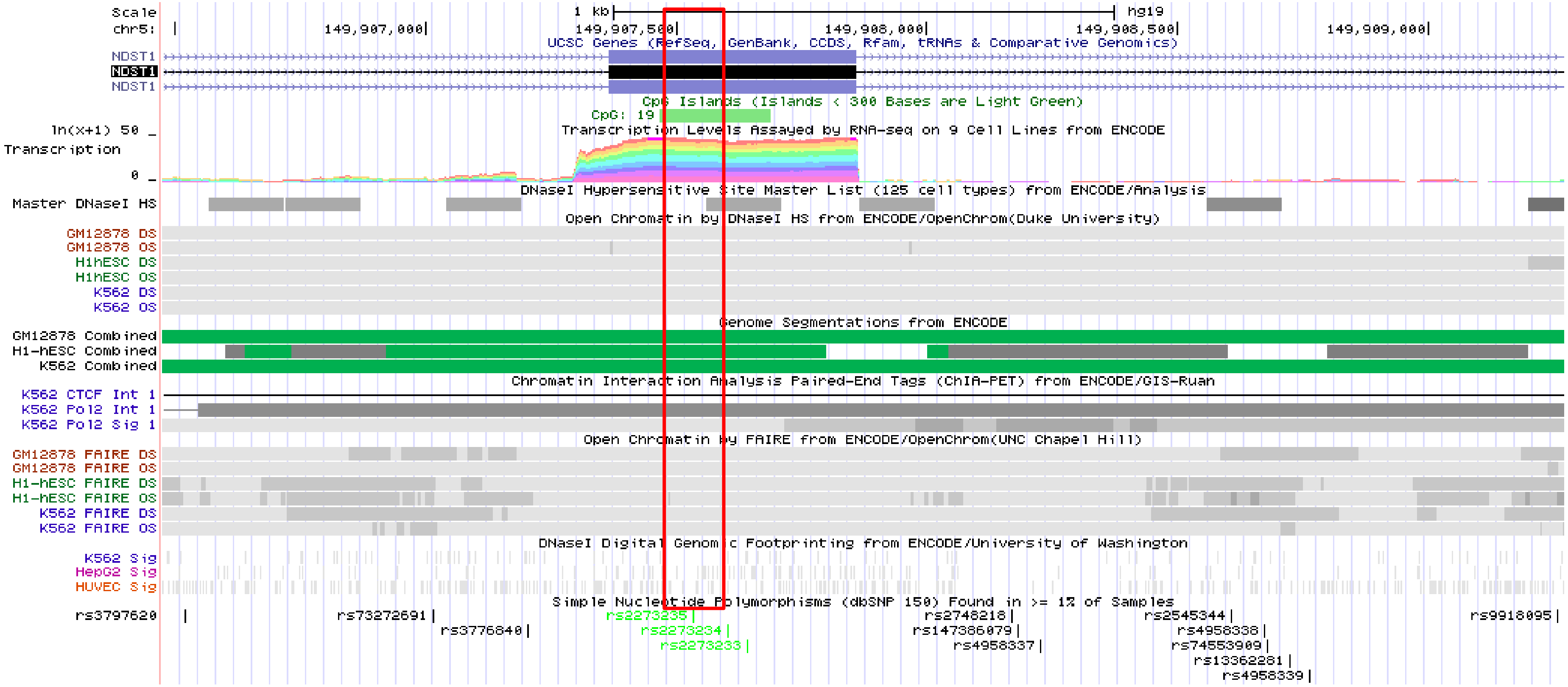

Figure S3 The genomic region of rs2273235

The synonymous m^{6}A-SNP rs2273235 locates in a CpG island (CpG: 19) and closes to DNase I hypersensitive sites.
